# Prognostic Impact of Interleukin-27 in Peripheral Artery Disease

**DOI:** 10.3390/life15111768

**Published:** 2025-11-18

**Authors:** Nataša Kokalj, Borut Jug

**Affiliations:** Department of Vascular Diseases, University Medical Centre Ljubljana, 1000 Ljubljana, Slovenia

**Keywords:** peripheral artery disease, interleukin-27, endovascular revascularization, major adverse cardiovascular events, major adverse limb events

## Abstract

Atherosclerosis is a progressive arterial disease characterized by chronic inflammation, with interleukin-27 (IL-27) implicated as both a pro- and anti-inflammatory cytokine. This prospective cohort study evaluated association of circulating IL-27 levels in peripheral artery disease patients undergoing elective endovascular revascularization, with major adverse cardiovascular events (MACE) and major adverse limb events (MALE) over a median follow-up of 311 days. Elevated IL-27 levels were significantly associated with increased risk of MACE and MALE in unadjusted analyses. After adjusting for established cardiovascular and PAD risk factors, IL-27 remained an independent predictor of MACE (HR 2.95; *p* = 0.039), but not MALE. These findings indicate that elevated IL-27 levels are associated with unfavourable long-term prognosis.

## 1. Introduction

Atherosclerosis is a progressive disease affecting large and medium-size arteries, which is characterized by the buildup of lipid-rich, fibrocellular lesions; i.e., plaque. Atherosclerotic vascular disease involves a complex process of lipoprotein accumulation, oxidation, and cell proliferation, wherein inflammation plays a crucial role. Local inflammation, including inflammatory cell (macrophage) migration and activation, plays a pivotal role in the pathophysiology of plaque formation, whereas systemic cytokine-mediated inflammatory pathways are involved in the initiation and progression of atherosclerosis, as well as plaque destabilization and atherothrombotic risk [1]. There are several pro-inflammatory cytokines with proven role in systemic atherosclerosis, including IL-27, IL-32, galectin-3 [2,3,4].

Interleukin-27 (IL-27) is an important cytokine with anti-inflammatory and pro-inflammatory properties [2]. The role of IL-27 is not dominantly pro-inflammatory; its role is rather context-dependent: it may initiate and promote inflammation but also plays a critical role in resolution of inflammation. It induces production of pro-inflammatory IL-1 and TNF-alpha, and inhibits the production of Th2 cytokines; its primary anti-inflammatory role is manifested in induction of production of anti-inflammatory interleukin-10 [5,6,7,8,9]. Recent studies on cardiomyocytes and vascular endothelium have demonstrated mechanisms through which IL-27 could potentially modulate atherosclerosis [10]. Upregulation of the IL-27 receptor was observed in the atherosclerotic plaques, whereas observational cross-sectional studies have shown that circulating IL-27 plasma levels are elevated in patients with atherosclerotic vascular disease, including patients with acute and chronic coronary syndromes [11] and patients with carotid atherosclerosis [12,13]. A recent prospective study in patients after myocardial infarction and in patients undergoing coronary angiography has also shown a prognostic role of IL-27 in patients with coronary artery disease [14]. Conversely, in patients with peripheral artery disease, the role of IL-27 has not been studied yet.

Thus, the aim of our study was to evaluate prognostic role of IL-27 in patients with peripheral arterial disease undergoing elective endovascular revascularization. We hypothesized that elevated circulating IL-27 levels would be associated with increased risk of major adverse cardiovascular events (MACE) and major adverse limb events (MALE) in patients with peripheral artery disease.

## 2. Materials and Methods

This prospective observational cohort study was conducted on consecutive patients with peripheral arterial disease, admitted at Department of Vascular Diseases at Universal Medical Centre Ljubljana for peripheral endovascular revascularization. Inclusion criteria were age ≥ 18 years and a documented index revascularization (including first endovascular procedure and also patients with subsequent endovascular procedure).

Clinical data on baseline characteristics were collected, including age, sex, limb involved, clinical stage, and ankle–brachial index. Comorbidities were assessed as binary or categorical variables: diabetes mellitus (DM), cerebrovascular disease (CVD), coronary artery disease (CAD), chronic kidney disease (CKD), heart failure (HF).

Data on current or past smoking status, the use of cardiovascular risk reduction medications (including statins, acetylsalicylic acid, angiotensin-converting enzyme inhibitors (ACEi)/angiotensin II receptor blockers (ARB)) and antithrombotic therapy at discharge were collected. Prior to endovascular procedure, peripheral blood samples were drawn 24 h from admission, fasting, at 7 a.m. Fasting blood samples were obtained from each participant by venipuncture according to the standard procedure and collected in 4 mL vacuum tube containing clot activator. Serum was prepared by centrifugation at 2000× *g* for 20 min. Immediately after centrifugation, serum was aliquoted and stored at ≤−70 °C until analysis. Serum concentration of IL-27 was measured with xMAP© technology using magnetic beads coupled with specific antibodies (all R&D Systems, Minneapolis, MN, USA) on a MagPix instrument (Luminex Corporation, Austin, TX, USA). Serum concentrations of creatinine levels, estimated glomerular filtration rate and IL-27 were assessed.

Exclusion criteria were chronic kidney disease stages IV and V (defined by estimated glomerular filtration rate < 30 mL/h), aborted endovascular procedure (due to clinical circumstances) and acute infection up to 6 weeks prior to admission.

After discharge, patients were followed up by clinical examination 3, 6, and 12 months after the procedure and yearly thereafter. At regular check-up examinations, data on clinical status, peripheral arterial disease symptoms, and ankle–brachial index were collected.

Time-to-event endpoints were derived for the following outcomes:

Major adverse cardiovascular events (MACE): (a) first occurrence of myocardial infarction (MI), (b) ischemic stroke/TIA (CVD), or (c) cardiovascular death.

Major adverse limb events (MALE): (a) first occurrence of major amputation due to critical ischemia, (b) progression to or relapse of chronic limb threatening ischemia, (c) ipsilateral endovascular re-interventions (urgent or elective). Endpoint adjudication was performed by two cardiovascular specialists, with a third cardiovascular specialist providing definite adjudication in case of discordant appraisal.

### Statistical Analysis

Baseline characteristics were summarized using means or medians (with SD/IQR) and proportions for continuous and categorical variables, respectively. Summary tables were created, and subgroup comparisons were performed for MALE and MACE strata using chi-square or Fisher’s exact test for categorical variables, and *t*-tests or Wilcoxon rank-sum tests for continuous variables, as appropriate.

To assess the prognostic relevance of IL-27, patients were stratified into quartiles based on the empirical distribution of biomarkers (<125, 125–225, 226–305, >305 ng/mL). Quartile groups were assigned using quantile-derived thresholds. Age was categorized into clinically meaningful strata: ≤60, 61–70, 71–80, and >80 years. Smoking and diabetes were further collapsed into binary variables (current smoking and any diabetes, respectively) for multivariable modeling. Rutherford stage was collapsed to a three-level factor: 2 (moderate claudication), 3 (severe claudication), and ≥4 (critical limb ischemia (CLI) were defined as resting ischemic limb pain or the presence of ischemic ulcers.

For missing follow-up dates, we imputed the control date as 12 months after the index procedure. Time-to-event calculations were performed in days.

Logistic regression models were constructed to evaluate the association between IL-27 quartiles and the risk of binary 1-year outcomes (i.e., amputation, limb revascularization, MI/unplanned coronary revascularization, cerebrovascular event, CV death, all-cause death). Multivariable models included pre-specified confounders: age, sex, CLI, CAD, CVD, diabetes, and smoking. Model fit and assumptions were verified using standard diagnostic procedures, and odds ratios (ORs) with 95% confidence intervals (CIs) were reported.

Time-to-event analyses were performed using Cox proportional-hazards models. Separate models were fitted for MACE and MALE using quartiles of IL-27 as primary predictors. Proportional-hazards assumptions were assessed using Schoenfeld residuals. Hazard ratios (HRs) with 95% CIs were reported. Kaplan–Meier curves stratified by biomarker quartiles were generated, and differences between curves were evaluated using log-rank tests with *p*-values annotated on plots.

All analyses were performed in R version 4.3.1. A two-tailed *p*-value < 0.05 was considered statistically significant.

## 3. Results

### 3.1. Enrollment and Follow-Up

A total of 489 patients, hospitalized during a period spanning from 1 January 2020 to 30 March 2022, were included in our study. A total of 15 patients (3.1%) were excluded from the analysis (1 because of chronic kidney disease stage V, 6 due to an aborted endovascular procedure); 8 patients (1.6%) were lost to follow up (alive according to vital status query, but we were unable to verify MACE or MALE components).

### 3.2. Baseline Patient Characteristics

Baseline patient characteristics are represented in Table 1. Mean age of all patients was 73 years, and 166 patients were female (Table 1).

### 3.3. Primary Outcome

During a median of 311 days (time to first event or censoring), 114 (24%) patients went on to experience MALE, and 48 (10%) experienced MACE. Subgroup comparisons for MALE and MACE are represented in Table 2 and Table 3, respectively.

### 3.4. Survival Analysis

In unadjusted models, patients in the highest IL-27 quartile (Q4) had a significantly increased risk of both MACE (HR 7.63, 95% CI 2.93–19.82, *p* < 0.001) and MALE (HR 1.31, 95% CI 0.79–2.16, *p* = 0.004). After adjusting for known cardiovascular and PAD risk factors, the association between IL-27 Q4 and MACE remained statistically significant (HR 2.95, 95% CI 1.06–8.22, *p* = 0.039); however, the association between IL-27 and MALE was attenuated and no longer statistically significant (HR 0.97, 95% CI 0.56–1.70, *p* = 0.926). The results are represented in Table 4 and Table 5, respectively.

**Figure 1:**
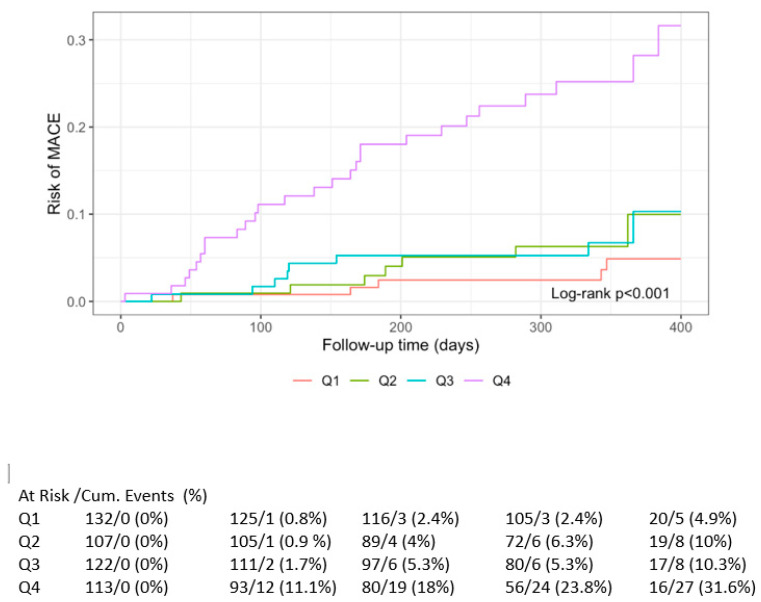
Kaplan–Meier curve for IL-27 and risk of MACE.

**Figure 2:**
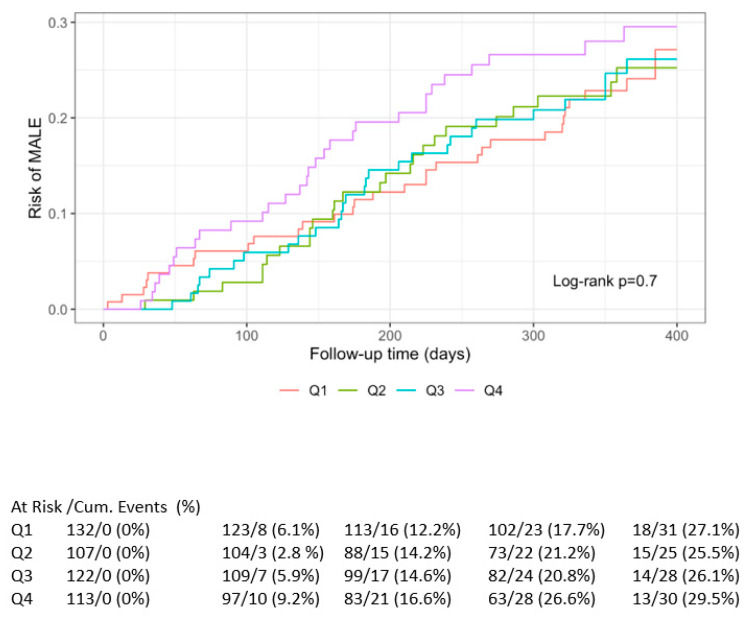
Kaplan–Meier curve for IL-27 and risk of MALE.

Kaplan–Meier curves for MACE and MALE are represented in Figure 1 and Figure 2, respectively.

## 4. Discussion

Circulating plasma levels of IL-27 represent a prognostic predictor in patients with peripheral artery disease. In our patient cohort, elevated IL-27 levels were associated with major long-term clinical outcomes, including myocardial infarction, stroke, major amputation, and coronary and peripheral revascularization. Overall, IL-27 levels were significant predictors of both MACE and MALE. However, after adjusting for traditional risk factors and co-morbidities—including age, sex, chronic limb-threatening ischemia, coronary artery disease, cerebrovascular disease (CVD), diabetes, and smoking—IL-27 levels independently predicted only MACE. Our study is the first to provide evidence on the prognostic role of IL-27 in peripheral artery disease. In this respect, our results align with evidence from observational studies in patients with coronary artery disease, which have shown that IL-27 are elevated in patients with atherosclerotic vascular disease and represent an independent predictor of future cardiovascular events in patients with coronary atherosclerosis [10,14,15,16].

The mechanistic role of IL-27 in atherosclerotic vascular disease remains elusive. IL-27 has been identified as both a pro-inflammatory and anti-inflammatory cytokine, with varying findings across different inflammatory conditions [1,2,17,18]. In atherosclerosis, IL-27 has been primarily identified as a pro-atherosclerotic cytokine, by promoting adhesion molecule upregulation in endothelial and CD4^+^ T cells [19,20]. Conversely, animal models have shown increased atherogenic potential in IL-27 receptor knocked-out mice and an antiatherogenic potential in recombinant IL-27 treated mice, suggesting an anti-atherosclerotic role of IL-27 [20,21,22,23]. Thus, studies with cell cultures and animal models paint a complex picture of the role of IL-27 in atherosclerosis, which remains to be elucidated. Conversely, elevated circulatory levels of IL-27 seem to represent an unfavorable finding in patients with atherosclerosis. Elevated IL-27 levels could plausibly be a marker of increased overall atherosclerotic disease burden, including in the context of PAD. IL-27 plays a role in chronic inflammation, which is central to atherosclerotic plaque development in multiple vascular territories, including peripheral arteries, coronary arteries, etc. Several case–control studies—but not all—have shown that circulating IL-27 levels are increased in patients with atherosclerotic vascular disease when compared to healthy controls, whereas data on the association between IL-27 levels and disease extent/severity are less straight forward [11,14,15,16]. Elevated circulating levels of IL-27 have also emerged as an unfavorable prognostic marker in critically ill patients, including patients with pancreatitis and pneumonia [24,25,26]. IL-27 gene polymorphisms have been linked to increased cardiometabolic risk, and preclinical and premature atherosclerosis. More importantly, in patients with cardiovascular disease, two studies have shown an association between IL-27 levels and unfavorable outcomes. One study in 524 patients with acute coronary syndrome has shown that patients with IL-27 levels in the highest tertile have a 2.7-fold higher risk of recurrent myocardial infarction or cardiovascular death when compared to patients with IL-27 in the lowest tertile [14]. A second study in 402 patients referred for coronary angiography (52% of whom had evidence of coronary artery disease) has shown that circulating IL-27 levels > 25 ng/mL have a 1.8-fold higher risk of future cardiovascular events [27]. Our study in patients with peripheral artery disease align with these two studies focusing on coronary atherosclerosis: in our analysis, circulating IL-27 levels in the highest tertile independently predicted a 3.6-fold increase in the risk of major cardiovascular events. Conversely, major limb events, while associated with increased IL-27 levels, could not be predicted by IL-27 after multivariate adjustment. Major cardiovascular events are driven primarily by systemic atherosclerotic burden and plaque instability, both of which are strongly linked to chronic vascular inflammation. Major limb events are complications of peripheral artery disease, which can be caused not only by chronic vascular inflammation but also by several other factors, such as severity of arterial stenosis/occlusion or microvascular disease. In our cohort, there was a higher proportion of MALE among patients with complex lesions (femoropopliteal and distal). This reflects more advanced atherosclerotic disease with multi-segment arterial involvement. Complex lesions are more challenging for revascularization and more prone to reduced long-term patency, which leads to restenosis and the need for repeat revascularization. In addition, such patients have often other comorbidities, such as diabetes mellitus and chronic kidney disease, which can further compromise tissue perfusion and lead to amputation.

### Limitations

Our study has some limitations. Firstly, we included patients with peripheral artery disease undergoing invasive diagnostic and/or revascularization procedures (Fontaine stages IIB–IV), representing a high-risk subset of patients with peripheral artery disease. Secondly, ours was a single-centre study; although providing data from a major national referral centre for patients with advanced peripheral arterial disease, limitations inherent to single-centre observation need to be accounted for. Thirdly, our observational study provides evidence of association, but not necessarily causation, between IL-27 and clinically relevant outcomes in patients with peripheral artery disease. Fourthly, there was no available data on levels of CRP. Though we did exclude patients with known acute infection up to 6 weeks prior to admission, undiagnosed inflammatory disease could potentially interfere with IL-27 levels.

## 5. Conclusions

Ours is the first study to address the long-term prognostic impact of circulating IL-27 levels in patients with peripheral artery disease. Our results have shown that elevated IL-27 levels are associated with unfavourable long-term prognosis—i.e., a higher risk of major adverse limb and cardiovascular events. Our findings suggest that IL-27 could have clinical value in identifying patients with peripheral artery disease with adverse prognosis, who may benefit from more intensive follow-up and more aggressive risk management/secondary prevention strategies, including high-intensity regimens of statins and ACE inhibitors.

## Figures and Tables

**Table 1:** Baseline patient characteristics.

Characteristic	*N = 474*
Age (years) ^1^	73 (65, 81)
Age group	
≤60	53 (11%)
61–70	152 (32%)
71–80	150 (32%)
>80	119 (25%)
Sex (female)	166 (35%)
Rutherford stage ≥ 4	222 (47%)
Primary segment involvement	
Iliac	90 (19%)
Femoropopliteal	135 (28%)
Distal	79 (17%)
Complex (femoropopliteal and distal)	170 (36%)
Diabetes mellitus	224 (47%)
Cerebrovascular disease	112 (24%)
CVI/Transient ischaemic attack	77 (17%)
Revascularization of carotid arteries	16 (3%)
>50% stenosis of carotid arteries	19 (4%)
Coronary artery disease	153 (32%)
Chronic kidney disease	140 (30%)
Acetylsalicylic acid	388 (82%)
Additional antithrombotic drug	
Clopidogrel	331 (71%)
Ticagrelor	3 (0.6%)
Prasugrel	1 (0.2%)
Statin therapy	
Less potent statins	125 (28%)
Atorvastatin/Rosuvastatin	233 (50%)
Ezetimib/PCSK9 inhibitors	4 (1%)
Combination of antilipemic therapy	35 (7%)
ACEi/ARB	328 (69%)
Amputation	30 (6%)
Unplanned next revascularisation	14 (3%)
Coronary event	14 (3%)
Cerebrovascular event	14 (3%)
Cardiovascular death	33 (7%)
Death from any cause	70 (15%)
Major cardiovascular event	48 (10%)
Major limb event	114 (24%)

^1^ Median (IQR); n (%). ACEi—Angiotensin-Converting Enzyme inhibitors, ARB—Angiotensin II Receptor Blockers, PCSK9—Proprotein convertase subtilisin/kexin type 9.

**Table 2:** Comparison of MALE/No event.

Characteristic	No MALE, *N = 349* ^1^	MALE, *N = 140* ^1^	*p*-Value ^2^
Age (years)	73 (65, 80)	72 (65, 81)	0.7
Age group			0.5
≤60	43 (12%)	10 (8.8%)	
61–70	113 (31%)	39 (34%)	
71–80	118 (33%)	32 (28%)	
>80	86 (24%)	33 (29%)	
Sex (female)	128 (36%)	38 (33%)	0.7
Rutherford stage ≥ 4	152 (42%)	70 (61%)	<0.001
Primary segment involvement			0.072
Iliac	76 (21%)	14 (12%)	
Femoropoliteal	104 (29%)	31 (27%)	
Distal	61 (17%)	18 (16%)	
Complex (femoropopliteal and distal)	119 (33%)	51 (45%)	
Diabetes mellitus	162 (45%)	62 (54%)	0.080
Chronic kidney disease	94 (26%)	46 (40%)	0.004
Cerebrovascular disease	85 (24%)	27 (24%)	>0.9
Coronary artery disease	122 (34%)	31 (27%)	0.2
Acetylsalicilic acid	295 (82%)	93 (82%)	>0.9
P2Y12/rivaroxaban	251 (70%)	84 (74%)	0.4
Statin therapy	305 (85%)	92 (81%)	0.3
ACEi/ARB	260 (72%)	68 (60%)	0.011

^1^ Median (IQR); n (%), ^2^ Wilcoxon rank sum test; Pearson’s Chi-squared test; Fisher’s exact test. ACEi—Angiotensin-Converting Enzyme inhibitors, ARB—Angiotensin II Receptor Blockers.

**Table 3:** Comparison of MACE/No event.

Characteristic	No MACE, *N = 426 ^1^*	MACE, *N = 48* ^1^	*p*-Value ^2^
Age (years)	72 (65, 79)	81 (73, 86)	<0.001
Age group			<0.001
≤60	50 (12%)	3 (6.3%)	
61–70	145 (34%)	7 (15%)	
71–80	137 (32%)	13 (27%)	
>80	94 (22%)	25 (52%)	
Sex (female)	147 (35%)	19 (40%)	0.5
Rutherford stage ≥ 4	185 (43%)	37 (77%)	<0.001
Primary segment involvement			0.6
Iliac	84 (20%)	6 (13%)	
Femoropoliteal	121 (28%)	14 (29%)	
Distal	69 (16%)	10 (21%)	
Complex (femoropopliteal and distal)	152 (36%)	18 (38%)	
Diabetes mellitus	198 (46%)	26 (54%)	0.3
Chronic kidney disease	110 (26%)	30 (63%)	<0.001
Cerebrovascular disease	99 (23%)	13 (27%)	0.6
Coronary artery disease	130 (31%)	23 (48%)	0.015
Acetylsalicilic acid	354 (83%)	34 (71%)	0.037
P2Y12/rivaroxaban	306 (72%)	29 (60%)	0.10
Statin therapy	367 (86%)	30 (63%)	<0.001
ACEi/ARB	301 (71%)	27 (56%)	0.040

^1^ Median (IQR); n (%), ^2^ Wilcoxon rank sum test; Pearson’s Chi-squared test; Fisher’s exact test. ACEi—Angiotensin-Converting Enzyme inhibitors, ARB—Angiotensin II Receptor Blockers.

**Table 4:** Unadjusted Cox proportional-hazards model for IL-27.

	MACE			MALE		
Predictors	Estimates	CI	*p*	Estimates	CI	*p*
qIL 27 [Q2]	2.03	0.66–6.21	0.214	1.02	0.60–1.72	0.948
qIL 27 [Q3]	1.88	0.62–5.75	0.268	1.03	0.62–1.72	0.902
qIL 27 [Q4]	7.63	2.93–19.82	**<0.001**	1.31	0.79–2.16	**0.004**
Observations	474			474		
R^2^ Nagelkerke	0.088			0.003		

CI—confidence interval, MACE—major adverse cardiovascular events, MALE—major adverse limb events.

**Table 5:** Cox proportional-hazards model for IL-27, adjusted for known cardiovascular and PAD risk factors.

	MACE			MALE		
Predictors	Estimates	CI	*p*	Estimates	CI	*p*
qIL 27 [Q2]	1.52	0.49–4.74	0.472	1.01	0.59–1.73	0.966
qIL 27 [Q3]	1.01	0.32–3.23	0.981	0.92	0.54–1.59	0.769
qIL 27 [Q4]	2.95	1.06–8.22	**0.039**	0.97	0.56–1.70	0.926
Age	1.06	1.02–1.10	**0.004**	1.00	0.98–1.02	0.830
Sex	0.93	0.48–1.81	0.835	0.80	0.51–1.24	0.319
CLI	2.97	1.32–6.67	**0.008**	2.05	1.29–3.26	**0.002**
Segment (Femoropopliteal)	1.10	0.40–2.98	0.857	1.41	0.74–2.70	0.295
Segment (Distal)	0.57	0.18–1.81	0.342	0.98	0.45–2.13	0.963
Segment (Complex femoropopliteal and distal)	0.45	0.15–1.35	0.154	1.38	0.71–2.70	0.345
CAD	1.60	0.89–2.89	0.119	0.75	0.49–1.15	0.187
CVD	1.22	0.63–2.36	0.545	1.12	0.72–1.74	0.616
Diabetes	1.27	0.68–2.39	0.457	1.12	0.75–1.67	0.579
Smoking	0.87	0.60–1.24	0.438	0.95	0.74–1.21	0.663
Observations	474			474		
R^2^ Nagelkerke	0.161			0.047		

CAD—coronary artery disease, CVD—cardiovascular disease, CI—confidence interval, CLI—critical limb ischemia, MACE—major adverse cardiovascular events, MALE—major adverse limb events.

## Data Availability

The raw data supporting the conclusions of this article will be made available by the authors on request.

## Abbreviations

The following abbreviations are used in this manuscript:

CAD	Coronary artery disease
CVD	Cardiovascular disease
IL	Interleukin
MACE	Major adverse cardiovascular events
MALE	Major adverse limb events
MI	Myocardial infarction
PAD	Peripheral artery disease
PTA	Percutaneus transluminal angioplasty
TIA	Transient ischaemic attack

## References

[1] Frostegård J., Ulfgren A.-K., Nyberg P., Hedin U., Swedenborg J., Andersson U., Hansson G.K. (1999). Cytokine expression in advanced human atherosclerotic plaques: Dominance of pro-inflammatory (Th1) and macrophage-stimulating cytokines. Atherosclerosis.

[2] Yoshida H., Nakaya M., Miyazaki Y. (2009). Interleukin 27: A double-edged sword for offense and defense. J. Leukoc. Biol..

[3] Onea H.-L., Homorodean C., Lazar F.-L., Negrea M.O., Calin T., Bitea I.C., Teodoru M., Nechita V.I., Olteanu A.L., Olinic D.-M. (2025). Galectin-3 Reflects Systemic Atherosclerosis in Patients with Coronary Artery Disease. Medicina.

[4] Damen M.S., Popa C.D., Netea M.G., Dinarello C.A., Joosten L.A. (2017). Interleukin-32 in chronic inflammatory conditions is associated with a higher risk of cardiovascular diseases. Atherosclerosis.

[5] Pflanz S., Timans J.C., Cheung J., Rosales R., Kanzler H., Gilbert J., Hibbert L., Churakova T., Travis M., Vaisberg E. (2002). IL-27, a heterodimeric cytokine composed of EBI3 and p28 protein, induces proliferation of naive CD4+ T cells. Immunity.

[6] Awasthi A., Carrier Y., Peron J.P.S., Bettelli E., Kamanaka M., Flavell R.A., Kuchroo V.K., Oukka M., Weiner H.L. (2007). A dominant function for interleukin 27 in generating interleukin 10-producing anti-inflammatory T cells. Nat. Immunol..

[7] Carl J.W., Bai X.-F. (2008). IL27: Its roles in the induction and inhibition of inflammation. Int. J. Clin. Exp. Pathol..

[8] Yoshida H., Hamano S., Senaldi G., Covey T., Faggioni R., Mu S., Xia M., Wakeham A.C., Nishina H., Potter J. (2001). WSX-1 is required for the initiation of Th1 responses and resistance to L. major infection. Immunity.

[9] Yoshimoto T., Yoshimoto T., Yasuda K., Mizuguchi J., Nakanishi K. (2007). IL-27 suppresses Th2 cell development and Th2 cytokines production from polarized Th2 cells: A novel therapeutic way for Th2-mediated allergic inflammation. J. Immunol..

[10] Posadas-Sánchez R., Pérez-Hernández N., Rodríguez-Pérez J.M., Coral-Vázquez R.M., Roque-Ramírez B., Llorente L., Lima G., Flores-Dominguez C., Villarreal-Molina T., Posadas-Romero C. (2017). Interleukin-27 polymorphisms are associated with premature coronary artery disease and metabolic parameters in the Mexican population: The genetics of atherosclerotic disease (GEA) Mexican study. Oncotarget.

[11] Jafarzadeh A., Nemati M., Rezayati M. (2011). Serum levels of interleukin-27 in patients with ischemic heart disease. Cytokine.

[12] Jafarizade M., Kahe F., Sharfaei S., Momenzadeh K., Pitliya A., Tajrishi F.Z., Singh P., Chi G. (2021). The role of interleukin-27 in atherosclerosis: A contemporary review. Cardiology.

[13] Gregersen I., Sandanger Ø., Askevold E.T., Sagen E.L., Yang K., Holm S., Pedersen T.M., Skjelland M., Krohg-Sørensen K., Hansen T.V. (2017). Interleukin 27 is increased in carotid atherosclerosis and promotes NLRP3 inflammasome activation. PLoS ONE.

[14] Grufman H., Yndigegn T., Gonçalves I., Nilsson J., Schiopu A. (2019). Elevated IL-27 in patients with acute coronary syndrome is associated with adverse ventricular remodeling and increased risk of recurrent myocardial infarction and cardiovascular death. Cytokine.

[15] Jin W., Zhao Y., Yan W., Cao L., Zhang W., Wang M., Zhang T., Fu Q., Li Z. (2012). Elevated circulating interleukin-27 in patients with coronary artery disease is associated with dendritic cells, oxidized low-density lipoprotein, and severity of coronary artery stenosis. Mediat. Inflamm..

[16] Miura K., Saita E., Suzuki-Sugihara N., Miyata K., Ikemura N., Ohmori R., Ikegami Y., Kishimoto Y., Kondo K., Momiyama Y. (2017). Plasma interleukin-27 levels in patients with coronary artery disease. Medicine.

[17] Bosmann M., Ward P.A. (2013). Modulation of inflammation by interleukin-27. J. Leukoc. Biol..

[18] Hunter C.A. (2005). New IL-12-family members: IL-23 and IL-27, cytokines with divergent functions. Nat. Rev. Immunol..

[19] Qiu H.N., Liu B., Liu W., Liu S. (2016). Interleukin-27 enhances TNF-α-mediated activation of human coronary artery endothelial cells. Mol. Cell. Biochem..

[20] Owaki T., Asakawa M., Morishima N., Hata K., Fukai F., Matsui M., Mizuguchi J., Yoshimoto T. (2005). A role for IL-27 in early regulation of Th1 differentiation. J. Immunol..

[21] Koltsova E.K., Kim G., Lloyd K.M., Saris C.J., Von Vietinghoff S., Kronenberg M., Ley K. (2012). Interleukin-27 receptor limits atherosclerosis in Ldlr-/- mice. Circ. Res..

[22] Hirase T., Hara H., Miyazaki Y., Ide N., Nishimoto-Hazuku A., Fujimoto H., Saris C.J.M., Yoshida H., Node K. (2013). Interleukin 27 inhibits atherosclerosis via immunoregulation of macrophages in mice. Am. J. Physiol. Heart Circ. Physiol..

[23] Xu W., Zhu R., Zhu Z., Yu K., Wang Y., Ding Y., Yu J., Tang H., Zeng Q., Zhong Y. (2022). Interleukin-27 ameliorates atherosclerosis in ApoE-/- mice through regulatory T Cell augmentation and dendritic cell tolerance. Mediat. Inflamm..

[24] Eric S., Zaric R.Z., Jevdjic J., Drakulic S.M., Stanojevic I., Vojvodic D., Arsenijevic P., Stojanovic B., Jakovljevic S., Markovic N. (2023). Interleukin 33, soluble suppression of tumorigenicity 2, interleukin 27, and galectin 3 as predictors for outcome in patients admitted to intensive care units. Open Med..

[25] Xu Z., Wang X.-M., Cao P., Zhang C., Feng C.-M., Zheng L., Xu D.-X., Fu L., Zhao H. (2022). Serum IL-27 predicts the severity and prognosis in patients with community-acquired pneumonia: A prospective cohort study. Int. J. Med. Sci..

[26] Xu F., Liu Q., Lin S., Shen N., Yin Y., Cao J. (2013). IL-27 is elevated in acute lung injury and mediates inflammation. J. Clin. Immunol..

[27] Saita E., Kishimoto Y., Ohmori R., Kondo K., Momiyama Y. (2024). Association between Plasma Interleukin-27 Levels and Cardiovascular Events in Patients Undergoing Coronary Angiography. J. Cardiovasc. Dev. Dis..

